# Identification of key genes and immune infiltration mechanisms in limb ischemia-reperfusion injury: a bioinformatics and experimental study

**DOI:** 10.3389/fimmu.2025.1491928

**Published:** 2025-05-09

**Authors:** Qiyun Shi, Taotao Tian, Yanfeng Li

**Affiliations:** ^1^ Medical Center of Cervical and Lumbar Pain (Tui Na Therapy), Luoyang Orthopedic-Traumatological Hospital Of Henan Province (Henan Provincial Orthopedic Hospital), Luoyang, China; ^2^ Medical Center of Microsurgery, Luoyang Orthopedic-Traumatological Hospital Of Henan Province (Henan Provincial Orthopedic Hospital), Zhengzhou, China

**Keywords:** limb ischemia-reperfusion injury, multi-tissue network analysis, immune microenvironment, bioinformatics modeling, therapeutic hub genes

## Abstract

**Aim of the study:**

To establish a cross-tissue bioinformatics model for identifying conserved key genes and immune infiltration mechanisms in ischemia-reperfusion injury (IRI) with experimental validation in limb IRI, including pharmacological targeting of the WNT5A/PLC pathway.

**Materials and methods:**

Transcriptomic data from CTGF-stimulated cardiac myocytes (GSE36073) were analyzed as a surrogate for limb IRI due to shared pathological mechanisms. Random forest, LASSO regression, algorithms identified feature genes, validated in a rat limb IRI model using RT-qPCR, and histology. Pharmacological inhibition (WNT5A inhibitor Box5, PLC inhibitor U-73122) was performed to assess pathway involvement. Immune cell infiltration patterns were analyzed via CIBERSORT.

**Results:**

From 169 differentially expressed genes (116 upregulated, 53 downregulated), machine learning identified four key genes (WNT5A, PLCG, ITPR1, CAMK2A), significantly upregulated in experimental limb IRI (P<0.01). Pharmacological inhibition confirmed their functional roles: Box5 and U-73122 treatment reduced expression of WNT5A and PLC versus IRI controls (P<0.05), showing IRI-induced muscle fiber disruption, edema, and inflammation. Immune analysis revealed myeloid polarization shifts (increased M1, decreased M2 macrophages; P<0.05). WNT5A correlated negatively with memory immune cells, while PLCG, ITPR1, and CAMK2A correlated with lymphocyte subpopulations.

**Conclusion:**

We identified a conserved molecular signature across cardiac and skeletal muscle IRI, with WNT5A/PLC pathway components as mechanistically validated therapeutic targets. Our cross-tissue bioinformatic approach, reinforced by pharmacological and histological evidence, provides a novel framework for IRI analysis when direct patient data are unavailable. Combined targeting of macrophage polarization and cellular activation the WNT5A/PLC axis may offer synergistic therapeutic potential.

## 1 Introduction

Ischemia-Reperfusion Injury (IRI) in the limb is a prevalent yet understudied condition in trauma surgery, where the restoration of blood flow following ischemia paradoxically exacerbates tissue damage (1) Unlike IRI in organs like the heart or brain, which has been extensively researched due to its critical implications in cardiovascular and neurological diseases (2), limb IRI remains less understood despite its frequency in clinical settings such as tourniquet use, vascular injuries, or limb replantation. This gap in knowledge is significant, as limb IRI can lead to severe outcomes, including prolonged recovery, chronic dysfunction, or amputation, underscoring the urgent need for effective prevention and treatment strategies. The pathological mechanisms of limb IRI share notable similarities with those observed in cardiac IRI, particularly the involvement of oxidative stress, inflammatory responses, and immune cell infiltration (3, 4). During ischemia, oxygen deprivation disrupts cellular metabolism, while reperfusion triggers a surge of reactive oxygen species (ROS) and inflammatory mediators, amplifying tissue injury through apoptosis, necrosis, and endothelial dysfunction (3–5). Studies suggest that immune cell infiltration, notably of neutrophils and macrophages, plays a pivotal role in driving inflammation and exacerbating damage in both limb and cardiac IRI (2, 6). However, the scarcity of comprehensive patient-derived data for limb IRI poses a significant challenge, limiting insights into its molecular underpinnings and hindering the development of targeted therapies.

Given these challenges, a cross-tissue analysis leveraging well-characterized cardiac IRI data offers a promising avenue to advance limb IRI research. The mechanistic overlap between limb and cardiac IRI—particularly in immune-mediated pathways—suggests that insights from cardiac studies could illuminate limb-specific processes (3, 4). Yet, current research often focuses narrowly on single organs, overlooking potential shared therapeutic targets across tissues. Traditional experimental approaches also struggle to capture the complexity of IRI’s multi-pathway nature, necessitating innovative strategies to integrate diverse biological data and identify key regulatory genes.

To address these limitations, this study employs model network analysis, a computational approach that integrates gene expression profiles, protein interaction networks, and signaling pathway data to construct comprehensive biological models (7). Widely applied in biomedical research, this method excels at uncovering disease-related gene interactions and predicting therapeutic targets (7). By combining cardiac and limb IRI datasets, we aim to bridge the data gap in limb IRI research and reveal shared and unique molecular drivers. Our approach not only compensates for the paucity of limb-specific data but also pioneers a cross-tissue perspective that could redefine IRI management.

**Figure 1:**
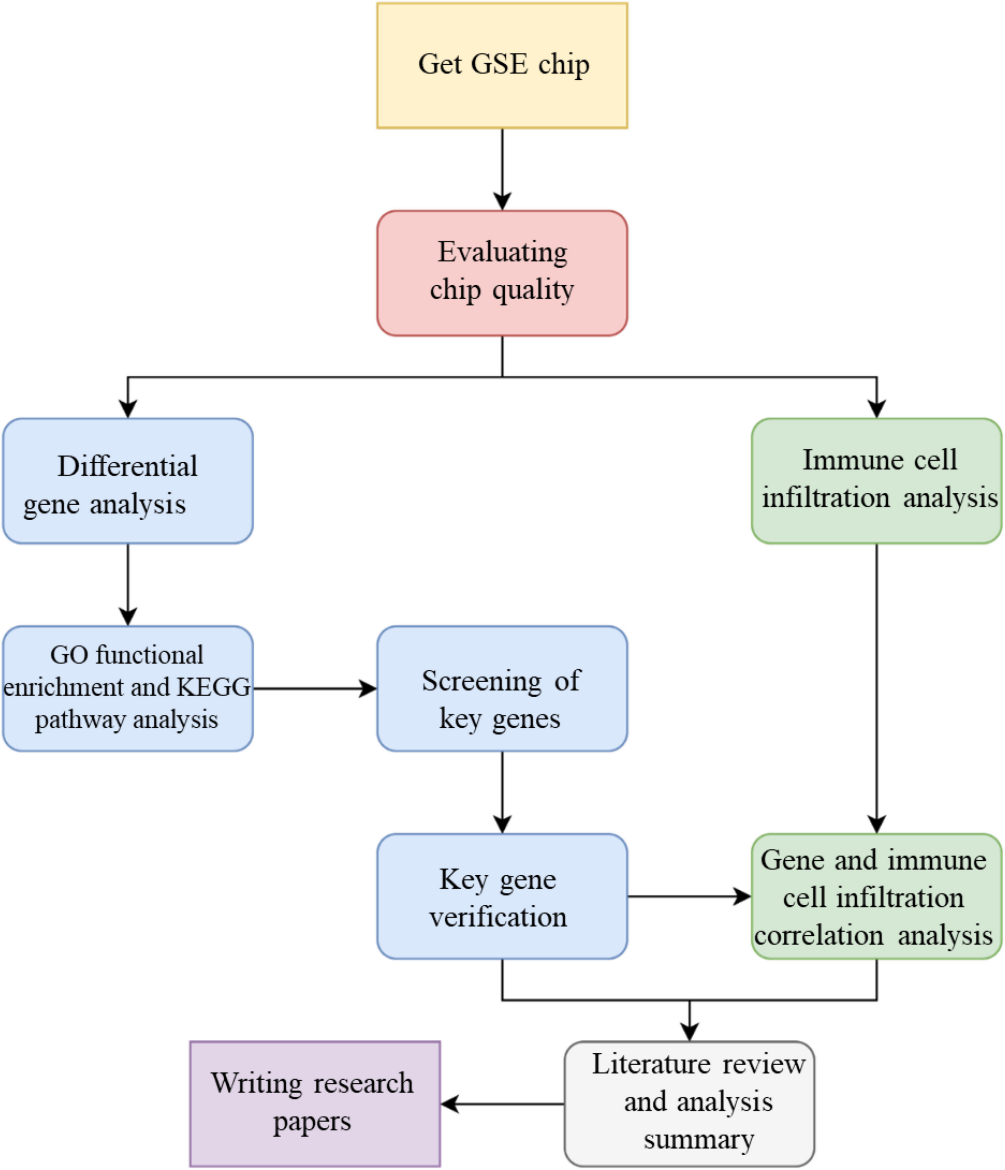
Technical route flow chart of this study.

The specific objectives of this study are: (1) to develop a cross-tissue biological network model of IRI by integrating cardiac and limb gene expression data, protein interactions, and immune-related signaling pathways; (2) to identify key genes and pathways common to limb and cardiac IRI, emphasizing immune infiltration regulators, using network analysis; (3) to validate the functional roles of these genes in limb IRI through *in vivo* mouse models, assessing their therapeutic potential; and (4) to quantify the contribution of immune cell infiltration to limb IRI progression and its correlation with identified gene targets. By harnessing a cross-tissue bioinformatics strategy, this study seeks to uncover novel molecular insights and therapeutic avenues, offering a significant step forward in understanding and mitigating limb IRI, the whole technical flow chart of this study displayed in **Figure 1**.

## 2 Materials and methods

### 2.1 Dataset selection

**Figure 2:**
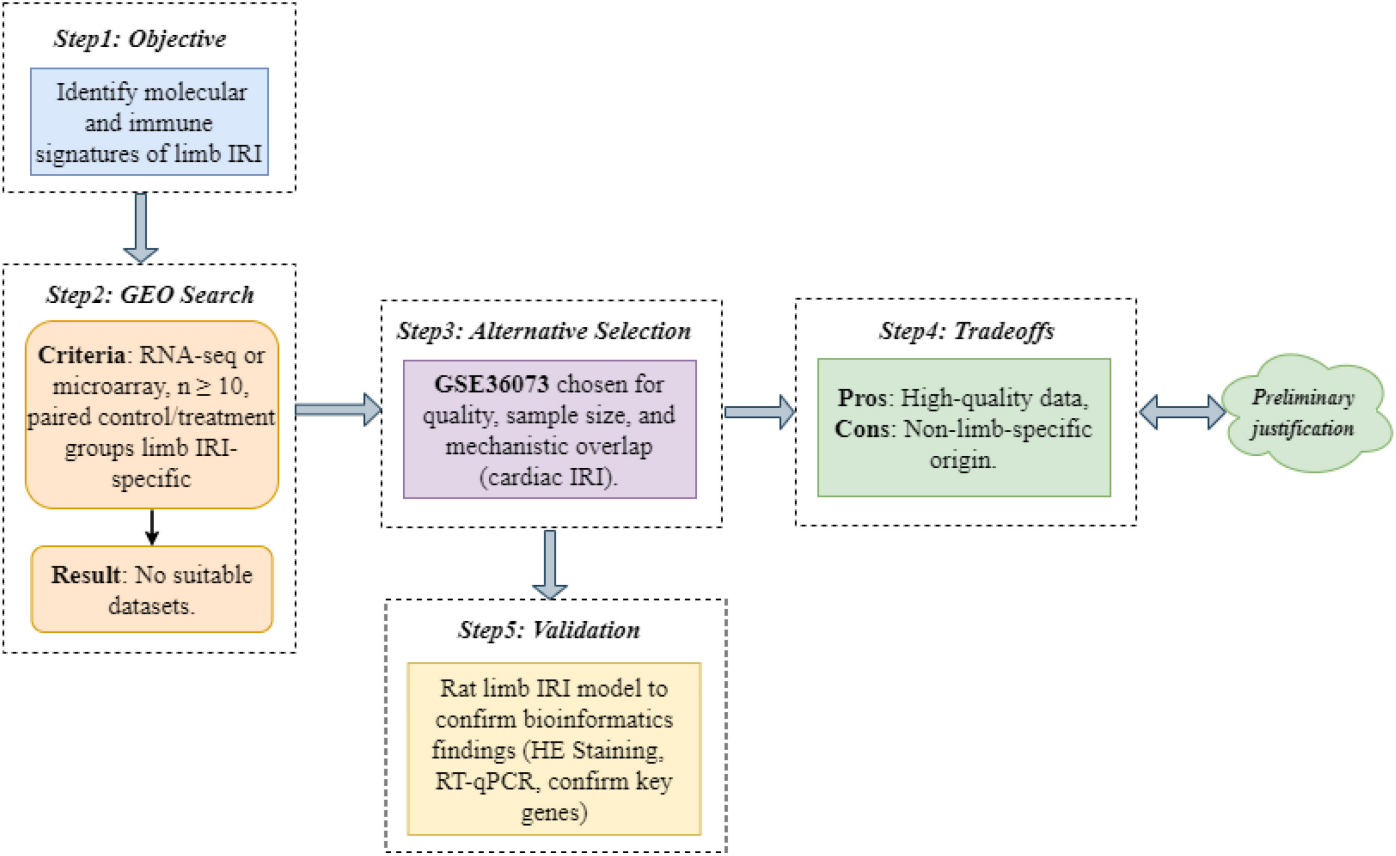
“Decision-making” process and tradeoffs for dataset selection in limb IRI analysis.

We aimed to identify molecular and immune signatures of limb IRI using high-throughput transcriptomic data. An exhaustive Gene Expression Omnibus (GEO) database (https://www.ncbi.nlm.nih.gov/geo/) search for limb IRI datasets (criteria: RNA-seq or microarray, n ≥ 10, paired control/treatment groups) yielded no suitable candidates. Consequently, we selected GSE36073, which profiles CTGF-stimulated adult cardiac myocytes, based on its high data quality (n = 12 samples), adequate sample size, and mechanistic overlap with limb IRI, including inflammatory responses and immune cell infiltration (3, 4). The GSE36073 dataset comprised 12 samples of primary adult mouse cardiac myocytes cultured under two conditions: 6 samples untreated (control group; GSM880719–GSM880723, GSM880729) and 6 samples stimulated with 200 nmol/L recombinant CTGF for 48 hours (model group; GSM880724–GSM880728, GSM880730). These samples, derived from C57BL/6 mice, were profiled using the Illumina MouseWG-6 v2.0 Expression BeadChip (GPL6887 platform) to investigate CTGF-induced transcriptional changes linked to IRI-related cardiac protection. Our decision-making process and tradeoffs are detailed in **Figure 2**. To validate findings, we constructed a rat limb IRI model (see Animal Experimental Validation below).

### 2.2 Identification of differentially expressed genes

Raw data from GSE36073 were first log-normalized using R software (version 4.3.1) to determine whether the expression matrix met the criteria for further analysis. A boxplot was then generated to check if the median expression levels of the samples were on the same level. Subsequently, clustering analysis and principal component analysis (PCA) were conducted to verify sample similarity. Finally, differential expression analysis was performed with a filter of |logFC| > 1 and adj.P < 0.05 to identify the differentially expressed genes (DEGs), both upregulated and downregulated.

### 2.3 GO functional enrichment and KEGG pathway analysis

GO and KEGG analyses were performed using the “clusterProfiler” package in R software. A significance threshold of P<0.05 was set for the enrichment results. The “ggplot2” package in R was used to visualize the results of GO functional enrichment and KEGG pathway analysis.

### 2.4 Machine learning for key gene selection

Two machine learning methods, Lasso regression and random forest, were used to identify key genes in IRI. First, the “randomForest” package in R was applied to select DEGs. To determine the optimal error rate and the number of trees for best stability, the error rate for 1 to 200 trees was calculated. The Gini coefficient method was used to rank the importance scores of the genes, and a random forest plot and gene importance plot were generated. The top 19 genes ranked by importance from the random forest analysis were selected. Next, the Lasso regression algorithm was used for cross-validation and key gene selection among the DEGs. The “venn” package in R was used to perform a Venn analysis, and the intersection of the gene sets obtained from the Lasso regression and the top 19 genes from the random forest algorithm was identified as the key genes for IRI.

### 2.5 Animal validation design and key gene verification

#### 2.5.1 Animal experiment design

Six healthy male SPF rats, aged 2 months and weighing (225 ± 25) g, were provided by the Henan Experimental Animal Center (Production license No.: SCXK(Yu)2022-0001). All rats were housed in the Animal Experiment Center of Henan University of Chinese Medicine under conditions of 60% humidity and 20°C. Animal use license number: SYXK(Yu)2021-0015. The experimental procedures strictly followed national or institutional regulations on the management and use of laboratory animals. The six rats were randomly divided into the model group and control group, with three rats per group. A modified method of external closure to block the femoral artery blood flow (8) was used, and the ischemic model was achieved by blocking the blood flow to the lower limbs for 4 hours. Upon successful modeling, a portion of the gastrocnemius muscle was fixed in formalin, dehydrated, embedded in paraffin, and sectioned for HE staining to observe muscle morphology and histopathological changes, confirming successful modeling. Total RNA was extracted from another portion of the muscle tissue using the Trizol method, cDNA was synthesized by reverse transcription, and key gene expression was amplified. The relative mRNA expression of key genes was detected by the 2-ΔΔCt method. The Trizol kit, reverse transcription kit, and RT-qPCR kit were provided by Baori Doctor Biotechnology (Beijing) Co., Ltd. Primers were synthesized by Sangon Biotech (Shanghai), with sequences shown in **Table 1**.

**Table 1:** Relevant primers.

Primer	Sequence5,-3,
WNT5A	ATGCGTGGAGCAGCGGCTTT
	TGGGAGGTGATGTTCTGGTG
PLCG	AGCAGTGACTCAGTGGAAGA
	TTGATGCCATCTGTTGAGGA
CAMK2A	AAGCAGCAGTGAGGGAGATG
	AGGGTGAGGAAGATGAAGGA
ITPR1	CGGATCCTAGTTTCTCCGTC
	AGGGTGAGGAAGATGAAGGA

#### 2.5.2 Statistical analysis

Statistical analysis was performed using SPSS 25.0 software. Data were expressed as mean ± standard deviation ( $\bar{x}$ ± s), and independent samples t-tests were used to analyze the differences in gene expression between the model group and the control group, with *P*<0.05 considered statistically significant. Graphs were plotted using GraphPad Prism 8.3.0 software.

### 2.6 Pharmacological validation through key pathway inhibition

To validate the involvement of the WNT5A/PLC signaling axis in IRI, we employed targeted pharmacological inhibition followed by molecular and histological analysis. This approach allowed us to systematically examine the functional consequences of disrupting specific nodes in the pathway. The following pharmacological agents and detection reagents were utilized: the WNT5A inhibitor Box5 (HY-123071, MedChemExpress), the PLC inhibitor U-73122 (S810156, Selleck), and specific antibodies against WNT5A (abs137051-50ug, Absin), PLCβ (abs149136-50ul, Absin), IP3R (ab108517-10ul, Absin), and CAMK II-α (50049S, CST).

Twenty adult male rats (225 ± 25) were randomly allocated into four experimental groups using a stratified randomization protocol:

1. Control Group (n=5): No intervention.
2. IRI Group (n=5): Subjected to 2 hours ischemia/4 hours reperfusion, an equal volume of normal saline was administered (intraperitoneally/intravenously) 30 minutes prior to ischemia.
3. Box5 pretreatment (n=5): Received 2 mg/kg Box5 i.p. 30 minutes pre-ischemia.
4. U-73122 treatment (n=5): Administered 1 mg/kg U-73122 i.v. at reperfusion onset.

Western Blot Protocol: Tissue collection was performed as previously described. Total protein was extracted using the RIPA method, and protein concentration was measured using a BCA protein quantification kit following the manufacturer’s instructions. Protein samples (25 μL per well) were loaded onto gels for electrophoresis, transferred to membranes, and subsequently blocked. Primary antibodies were incubated overnight at 4°C, followed by incubation with secondary antibodies for 90 minutes at room temperature. ECL chemiluminescence was detected using a Gel Doc XR+ imaging system. Relative protein expression was calculated by determining the ratio of target protein to Tubulin gray values using ImageJ software.

qPCR and HE staining were performed following previously described protocols and conducted statistical analysis and graphing as outlined earlier.

### 2.7 Immune cell content analysis

The CIBERSORT algorithm was used for correlation analysis of infiltrating immune cells, with perm=1000 and P<0.05 as the filtering conditions to obtain the relative content of 22 types of immune cells in the model and normal groups. Differences in immune cells between the two groups and the correlations among different immune cells were analyzed. Visualization was performed using the “corrplot” and “vioplot” packages in R.

### 2.8 Correlation analysis between identified genes and immune cell infiltration

The “corrplot” package in R was used to study the correlation between the identified potential biomarkers and immune cell infiltration levels, and the “ggplot2” package in R was used for visualization.

## 3 Results

### 3.1 Quality analysis of microarray data

**Figure 3:**
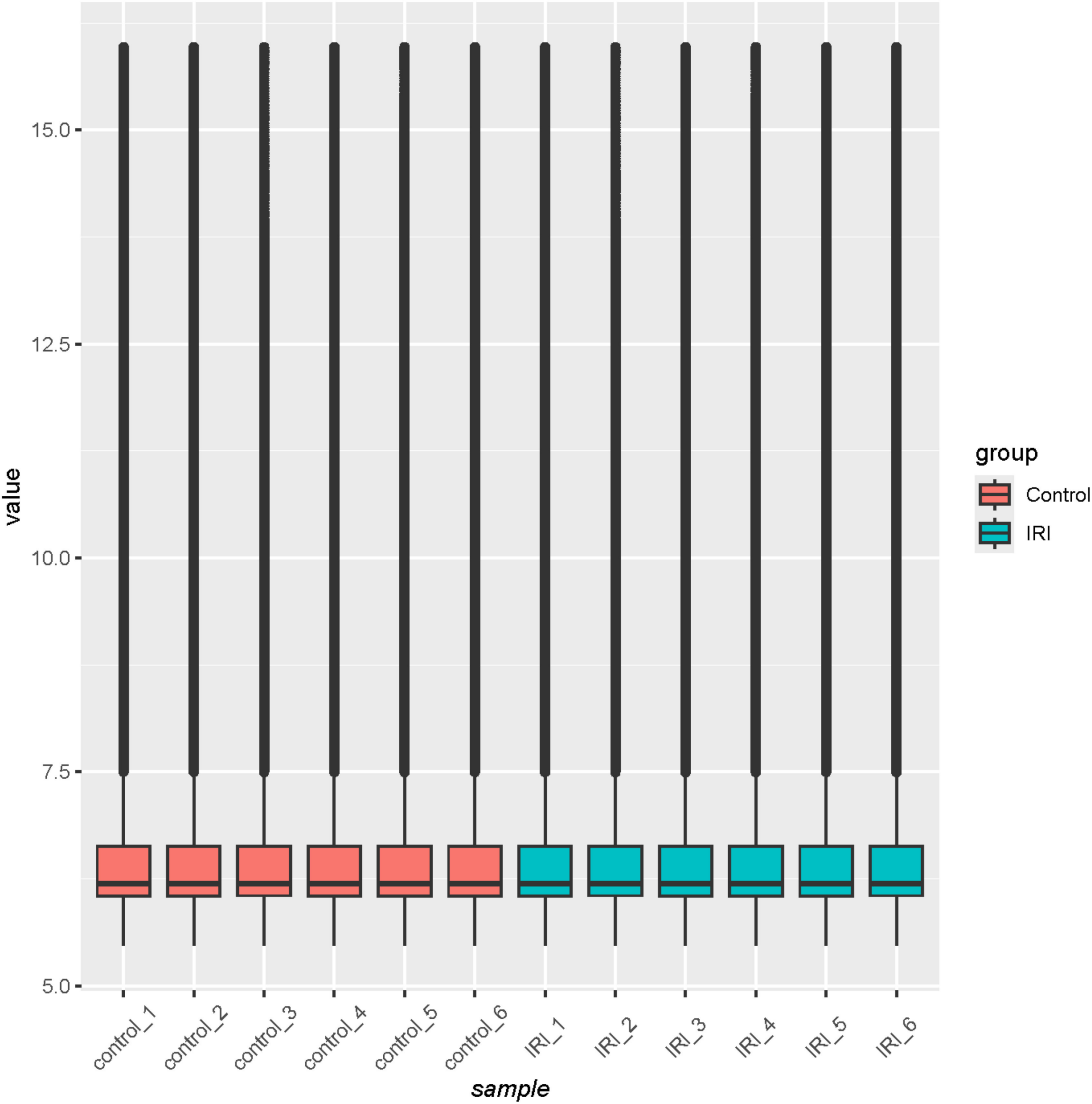
Boxplot after data normalization.

**Figure 4:**
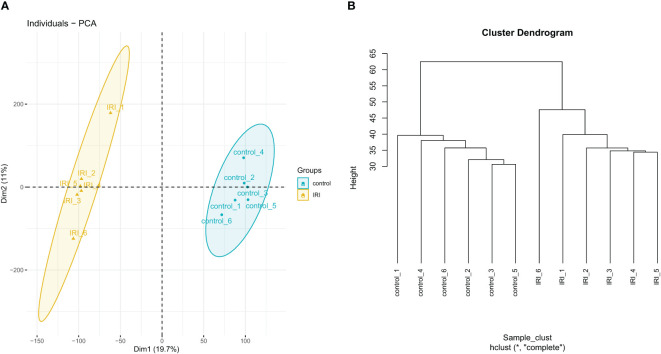
Principal component analysis plot **(A)** and clustering analysis plot **(B)**.

Quality analysis of the GSE36073 gene microarray data showed that the median expression levels of each sample were aligned on the same horizontal line in the boxplot (**Figure 3**), indicating consistent overall expression levels. Subsequent clustering analysis and principal component analysis (**Figures 4A, B**) confirmed that the data met the criteria for further analysis.

### 3.2 Acquisition of DEGs

**Figure 5:**
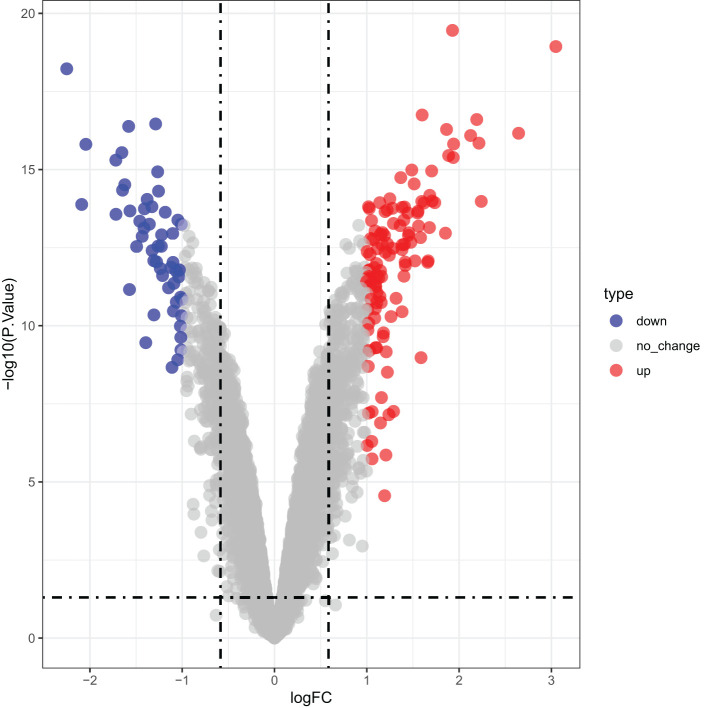
Volcano plot of differential gene expression.

Differential expression analysis identified 169 DEGs associated with IRI, including 116 upregulated and 53 downregulated genes in the CTGF-stimulated model group compared to the control, which was shown in a volcano plot in **Figure 5**.

### 3.3 GO and KEGG enrichment analysis

**Figure 6:**
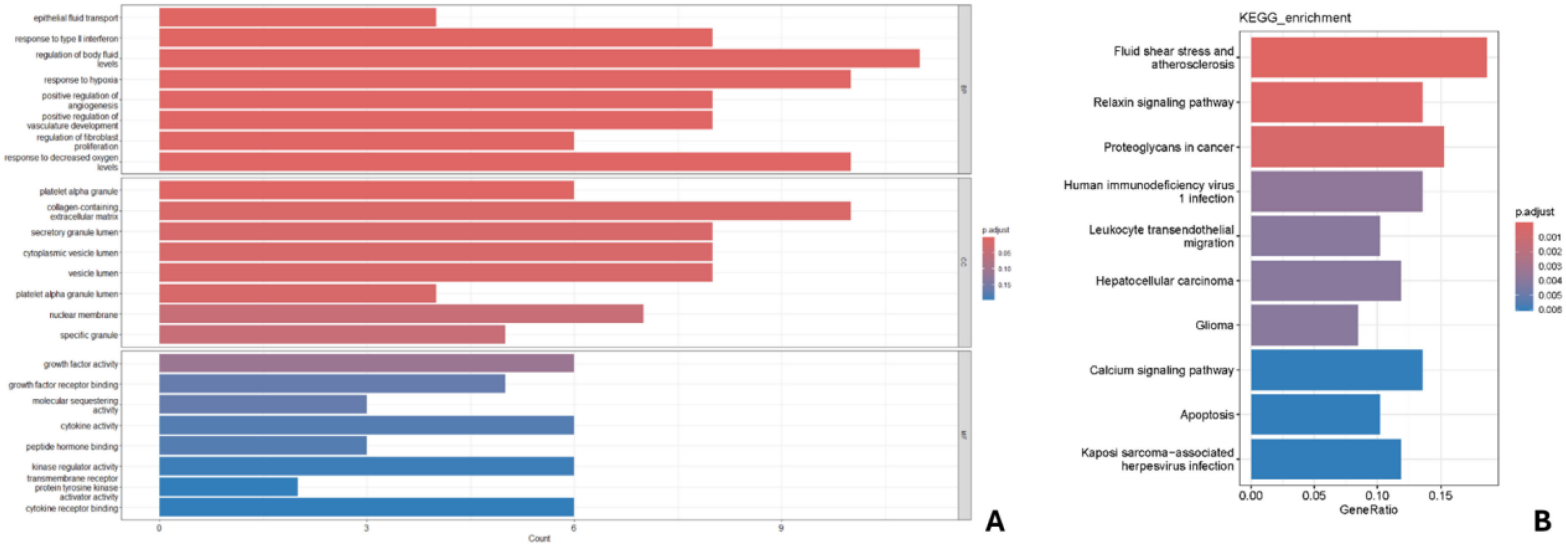
Bar chart of GO enrichment **(A)** and KEGG enrichment **(B)** analysis.

GO analysis of key genes (**Figure 6A**) indicated enrichment in biological processes such as epithelial fluid transport, response to type II interferon, regulation of fluid levels, response to hypoxia, and positive regulation of angiogenesis. Cellular components included platelets, extracellular matrix, secretory granules, vesicle lumen, and nuclear membrane; molecular functions included growth factor activity, molecular isolation activity, and cytokine activity. KEGG analysis identified 10 enriched pathways (**Figure 6B**), with the strongest correlations found in fluid shear stress and atherosclerosis, relaxin signaling, and calcium signaling pathways. Notably, calcium signaling is a current research hotspot, and other correlations were found with proteoglycans in cancer and apoptotic changes.

### 3.4 Machine learning for IRI key gene selection

**Figure 7:**
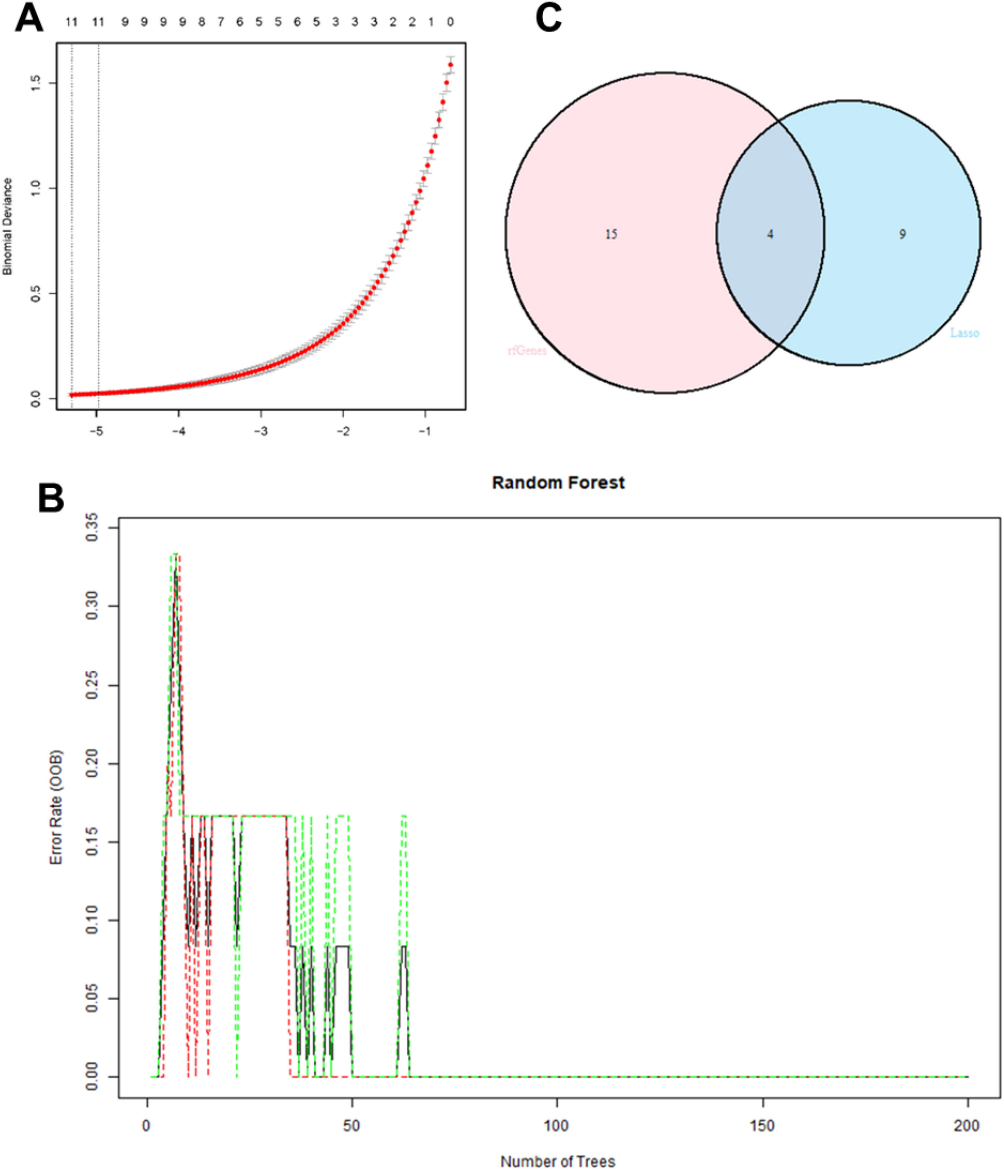
10-fold cross-validation in Lasso regression to obtain IRI characteristic genes **(A)**; cross-validation error calculation in the random forest algorithm **(B)**; Intersection of characteristic genes obtained from both machine learning algorithms **(C)**.

Through Lasso regression, 10-fold cross-validation was performed to obtain IRI characteristic genes (**Figure 7A**). In the random forest algorithm, the parameter “ntree” was set to 200 to calculate the cross-validation error (**Figure 7B**). The importance score for each gene was calculated based on the mean decrease in the Gini index, and the intersection of characteristic genes obtained from both machine learning algorithms yielded four representative IRI key genes (WNT5A, PLCG, ITPR1, CAMK2A) (**Figure 7C**). The list of key gene is in **Table 2**.

**Table 2:** Key genes selection from two machine learning methods.

Lasso regression	Random Forest
WNT5A	PLCG2
SAMD9L	FEZ1
PLCG2	4933402G07RIK
FEM1B	WNT5A
CLDN5	OSBPL3
GNGT2	CAMK2A
CAMK2A	NFATC1
LY6A	ITPR1
E130012P04RIK	IGFBP3
OASL2	JAZF1
ITPR1	DTNA
GDF15	GEMIN4
AQP1	GTSE1
	PIGO
	VEZF1
	HIST2H2BE
	E230029F23RIK
	CYB561

### 3.5 Experimental validation in rat limb IRI model

Bioinformatics analysis of GSE36073 predicted upregulation of WNT5A (logFC= 1.886944964, P.Value = 3.56E-16), PLCG (logFC= 2.191536846, P.Value = 2.50E-17), ITPR1 (logFC= 1.368207028, P.Value = 1.82E-15), and CAMK2A (logFC= 2.125159336, P.Value = 8.15E-17) and increased macrophage infiltration in IRI. In our rat limb IRI model, RT-qPCR confirmed WNT5A (p = 0.000103), PLCG (p = 0.0000418), ITPR1(p = 0.003078), and CAMK2A (p = 0.000103) upregulation compared to sham controls, consistent with in silico findings.

**Figure 8:**
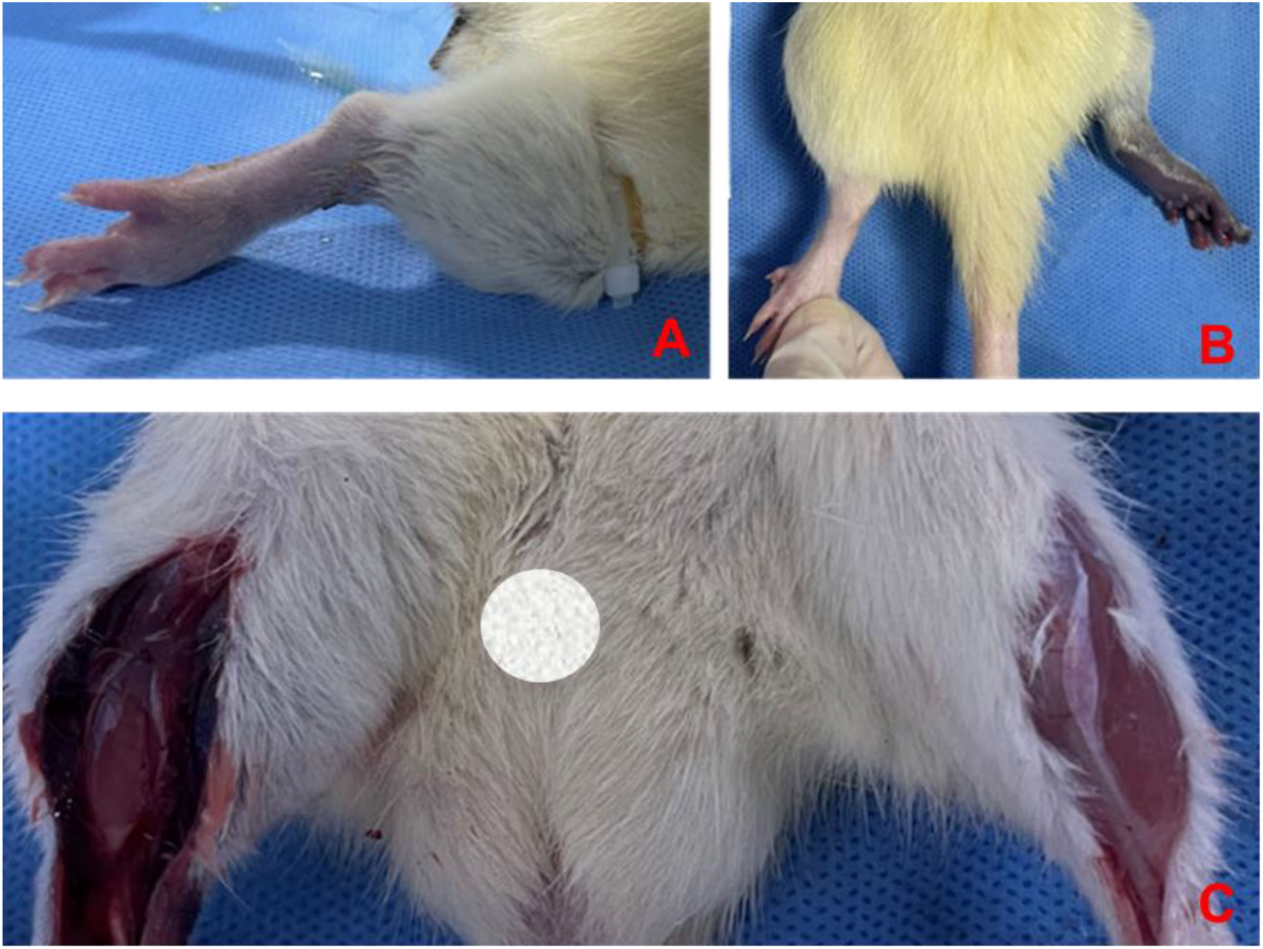
Changes in hind limb skin and muscle post-ischemia and reperfusion. Pre-modeling **(A)** shows rosy skin with normal temperature and no swelling, after 4 hours of ischemia **(B)** the skin appears cyanotic, cold, and significantly swollen, after 4 hours of reperfusion **(C)** normal rat muscle is firm, smooth, and evenly red without swelling or atrophy, while the IRI group gastrocnemius muscle is markedly swollen with uneven dark red appearance and hemorrhagic spots.

**Figure 9:**
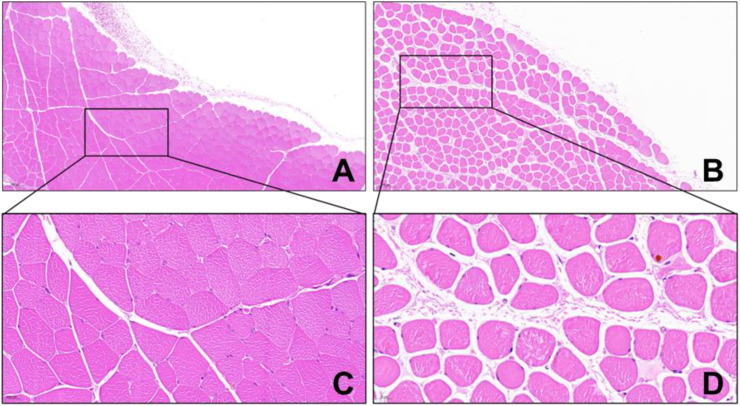
HE staining of rat gastrocnemius tissue (×100 in **A, B**, ×200 in **C, D**) (**A, C** Control Group; **B, D** IRI Group).

Before modeling, the hind limb skin of rats was rosy with normal skin temperature and no obvious swelling. After 4 hours of ischemia, the skin was cyanotic, cold, and significantly swollen (**Figures 8A, B**). After 4 hours of reperfusion, normal rat muscle appeared firm, smooth, and evenly red, without obvious swelling or atrophy. In the IRI group, the gastrocnemius muscle was markedly swollen, with uneven appearance and dark red color, and hemorrhagic spots on the surface (**Figure 8C**). Microscopic examination after staining showed: (1) Cell Morphology: Skeletal muscle fibers in the normal group were arranged neatly with regular morphology; in the IRI group, muscle fibers were irregular, with partial fibers broken or deformed. (2) Cell Structure: The cytoplasm appeared uniformly red without obvious pathological changes, and distinct sarcomere structures were visible in muscle fibers; in the IRI group, the cytoplasm showed varying degrees of uneven red staining, with blurred or absent muscle fiber structures, and swelling or vacuolation in some areas. (3) Intercellular Matrix: The normal group had less interstitial matrix, with tightly organized tissues, no significant inflammatory cell infiltration, and normal vascular distribution; in IRI model, significant inflammatory cell infiltration, mainly neutrophils and macrophages, was observed in the matrix, with perivascular edema, fibrin exudation, and coagulation within the tissue (**Figure 9**).

**Figure 10:**
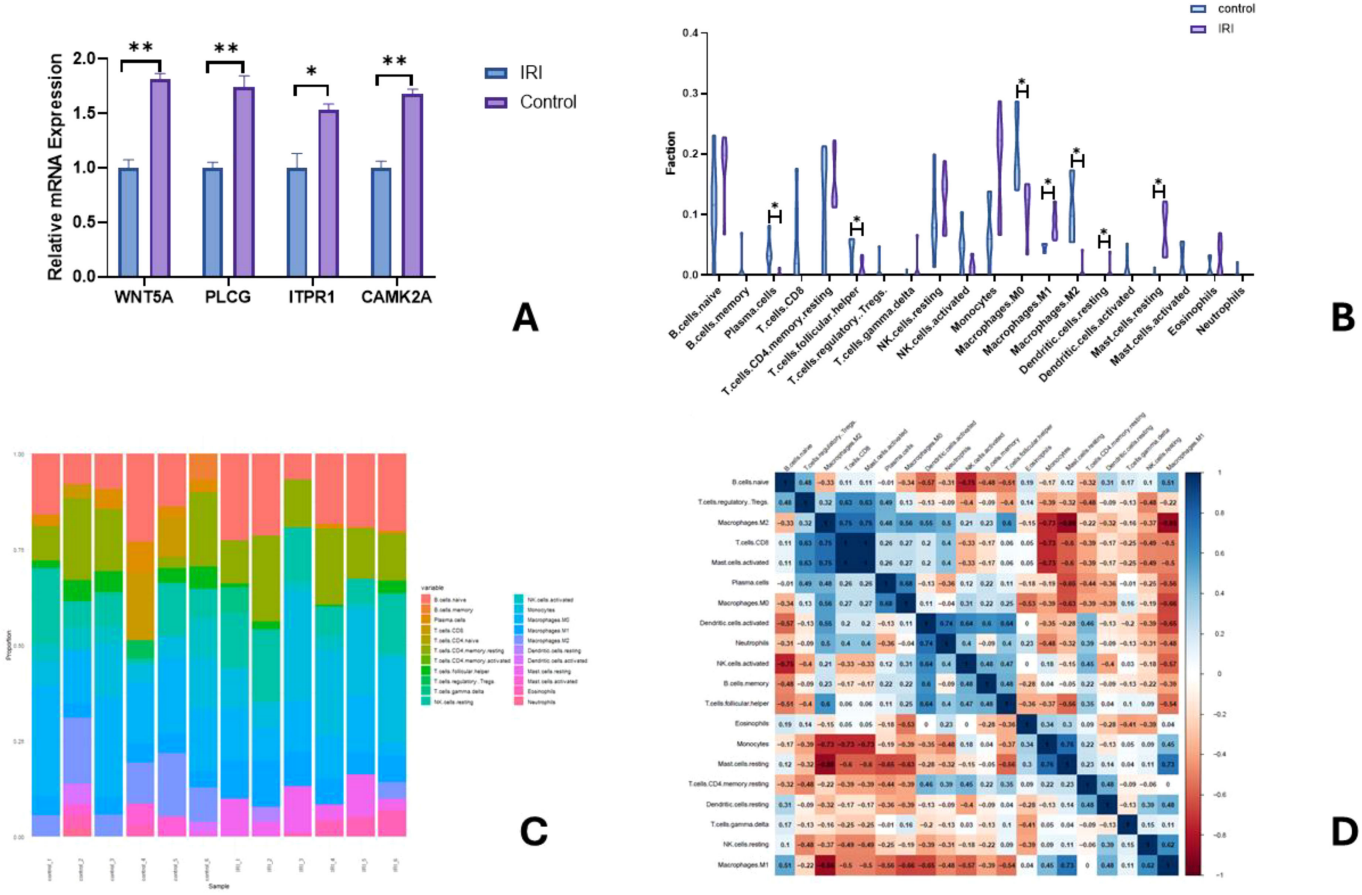
Relative mRNA expression of key genes in **A** (∗ indicates P<0.01, ∗∗ indicates P<0.001); Comparison of relative immune cell expression levels in **B** (Note: ∗ indicates P<0.01); Bar graph of immune cell infiltration proportions in **C**; Heatmap of immune cell correlation in **D**.

RT-qPCR was used to detect mRNA expression levels of IRI predictive genes. Experimental validation showed that the expression levels of WNT5A, PLCG, ITPR1, and CAMK2A were significantly upregulated in the IRI group compared to the control group (**Figure 10A**).

### 3.6 Pharmacological validation results

#### 3.6.1 Protein expression of WNT5A, PLC, IP3R, and CAMK II in skeletal muscle tissue

**Figure 11:**
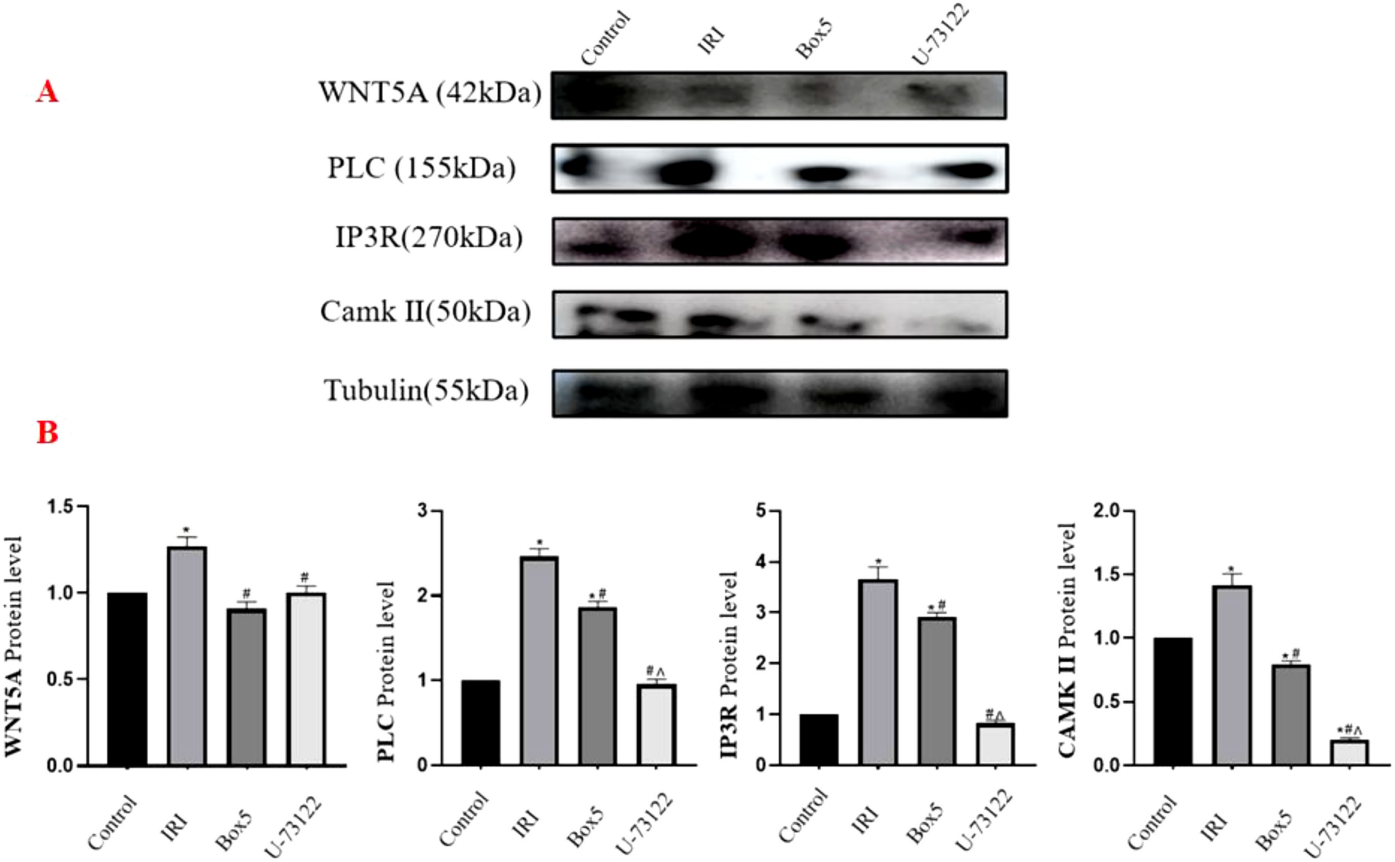
Protein expression of WNT5A, PLC, IP3R, and CAMK II in skeletal muscle tissue. **(A)** Western blot images. **(B)** Quantification of relative protein levels normalized to Tubulin. (Data are presented as mean ± SEM. *P<0.05 *vs* Control;# P<0.05 *vs* IRI;^ P<0.05 *vs* Box5).

As shown in the **Figure 11**, compared with the Control Group, the IRI Group exhibited significantly increased expression of WNT5A, PLC, IP3R, and CAMK II (p < 0.05). In the Box5 Group: WNT5A, PLC, IP3R, and CAMK II expression decreased significantly compared with the Model Group (p < 0.05). Compared with the Control Group, WNT5A showed no significant change, while PLC and IP3R were upregulated (p < 0.05), and CAMK II was downregulated (p < 0.05). In the U-73122 Group: Compared with the Control Group, WNT5A, PLC, and IP3R showed no significant changes, while CAMK II was downregulated (p < 0.05). Compared with the IRI Group, all four proteins (WNT5A, PLC, IP3R, CAMK II) were downregulated (p < 0.05). When comparing the U-73122 Group with the Box5 Group: WNT5A showed no significant difference, while PLC, IP3R, and CAMK II were further downregulated in the U-73122 Group (p < 0.05).

#### 3.6.2 mRNA expression of WNT5A, PLC, IP3R, and CAMK II in skeletal muscle tissue

**Figure 12:**
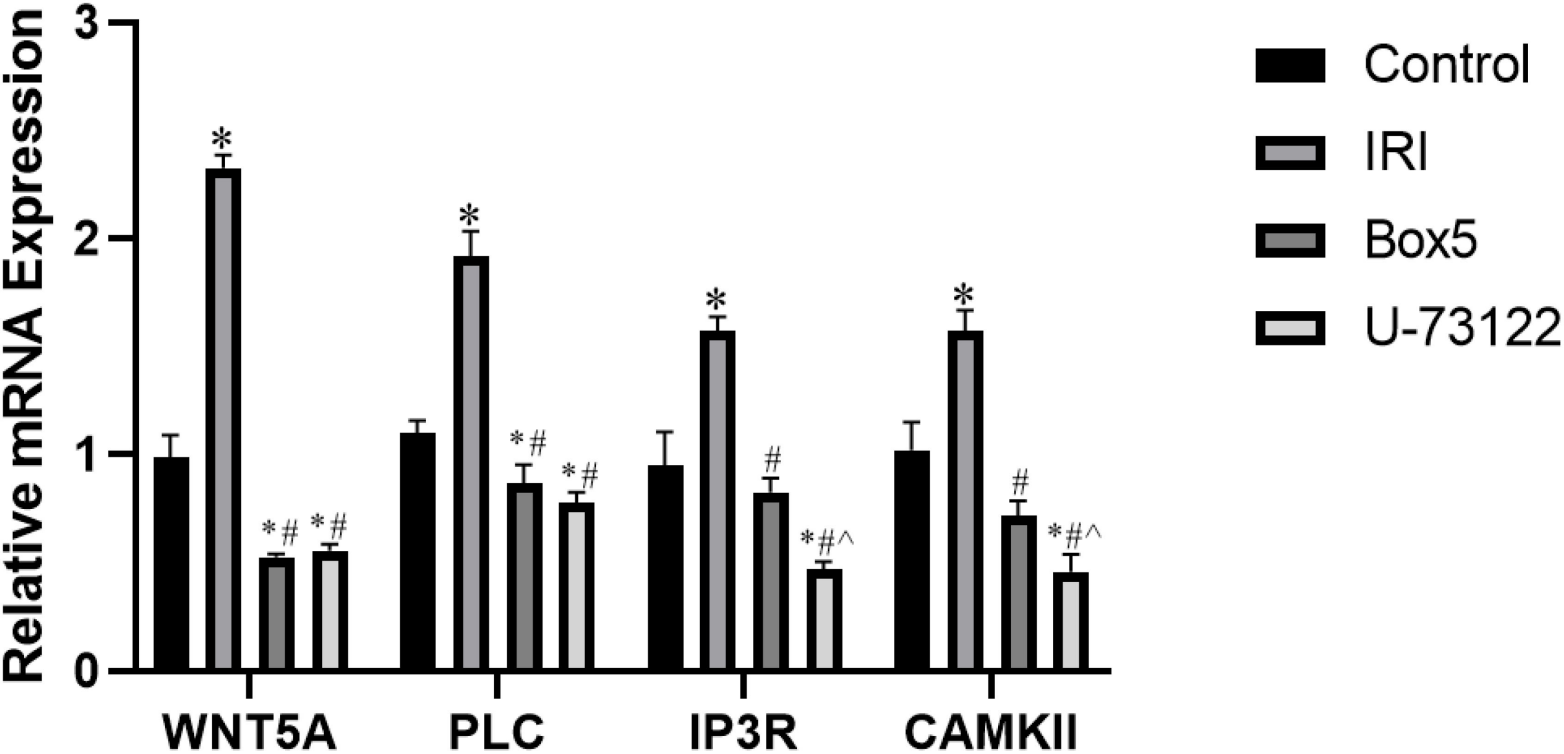
mRNA expression of WNT5A, PLC, IP3R, and CAMK II in skeletal muscle tissue. (Data are presented as mean ± SEM. *P<0.05 *vs* Control;# P<0.05 *vs* IRI;^ P<0.05 *vs* Box5).

As shown in the **Figure 12**: WNT5A: The IRI Group showed significantly higher expression than the Control Group, Box5 Group, and U-73122 Group (p < 0.001). Both the Box5 Group and U-73122 Group showed significantly lower expression than the Normal Control Group (p < 0.05). PLC: The Model Group showed significantly higher expression than the Control Group, Box5 Group, and U-73122 Group (p < 0.001). Both the Box5 Group and U-73122 Group showed significantly lower expression than the Control Group (p < 0.05). IP3R: The IRI Group showed significantly higher expression than the Control Group, Box5 Group, and U-73122 Group (p < 0.001). The U-73122 Group showed significantly lower expression than the Control Group and Box5 Group (p < 0.05). CAMK II: The IRI Group showed significantly higher expression than the Control Group, Box5 Group, and U-73122 Group (p < 0.001). The U-73122 Group showed significantly lower expression than the Control Group and Box5 Group (p < 0.05).

#### 3.6.3 HE staining results of skeletal muscle tissue

**Figure 13:**
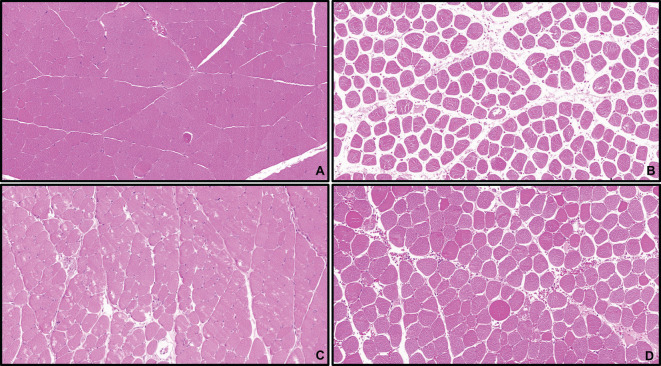
HE staining of skeletal muscle tissue.(×100) Control **(A)**, IRI **(B)**, Box5 **(C)** and U-73122 **(D)**.

As shown in the **Figure 13**: Control Group: Gastrocnemius muscle fibers appeared as long, cord-like structures with neat arrangement, clear architecture, and abundant sarcoplasm. Multiple nuclei were tightly attached to the inner surface of each muscle fiber, with uniform size and staining, and no abnormal changes were observed. IRI Group: Most muscle fibers were disrupted, with incomplete structures, irregular arrangement, thickening, edema, and widened inter-fiber gaps. Nuclei were sparse and scattered, with significant inflammatory infiltration. Box5 Group and U-73122 Group: Muscle fibers were slightly swollen and thinned, with relatively intact structures. Partial inter-fiber gaps were widened, and inflammatory infiltration was milder compared with the IRI Group. Pathological changes were significantly reduced compared with the IRI Group.

### 3.7 Immune cell infiltration results

A violin plot (**Figure 10B**) was used to visualize differences in immune cell infiltration among different tissue samples. The infiltration levels of plasma cells, T cells follicular helper, Macrophages M0, and Macrophages M2 were significantly decreased in the IRI group (P<0.05), while the levels of Macrophages M1, Dendritic cells resting, and Mast cells activated were significantly increased (P<0.05). The CIBERSORT deconvolution method was used to analyze the distribution changes of immune cells in the samples (**Figure 10C**). Positive correlations were observed between resting mast cells and monocytes (r=0.76), Macrophages M2 and T cells CD8 (r=0.75), Macrophages M2 and activated mast cells (r=0.75); negative correlations were found between resting mast cells and Macrophages M2 (r=-0.88), Macrophages M2 and Macrophages M1 (r=-0.86), and NK cells activated and B cells naive (r=-0.75) (**Figure 10D**).

### 3.8 Correlation analysis of gene potential biomarkers and infiltrating immune cells

**Figure 14:**
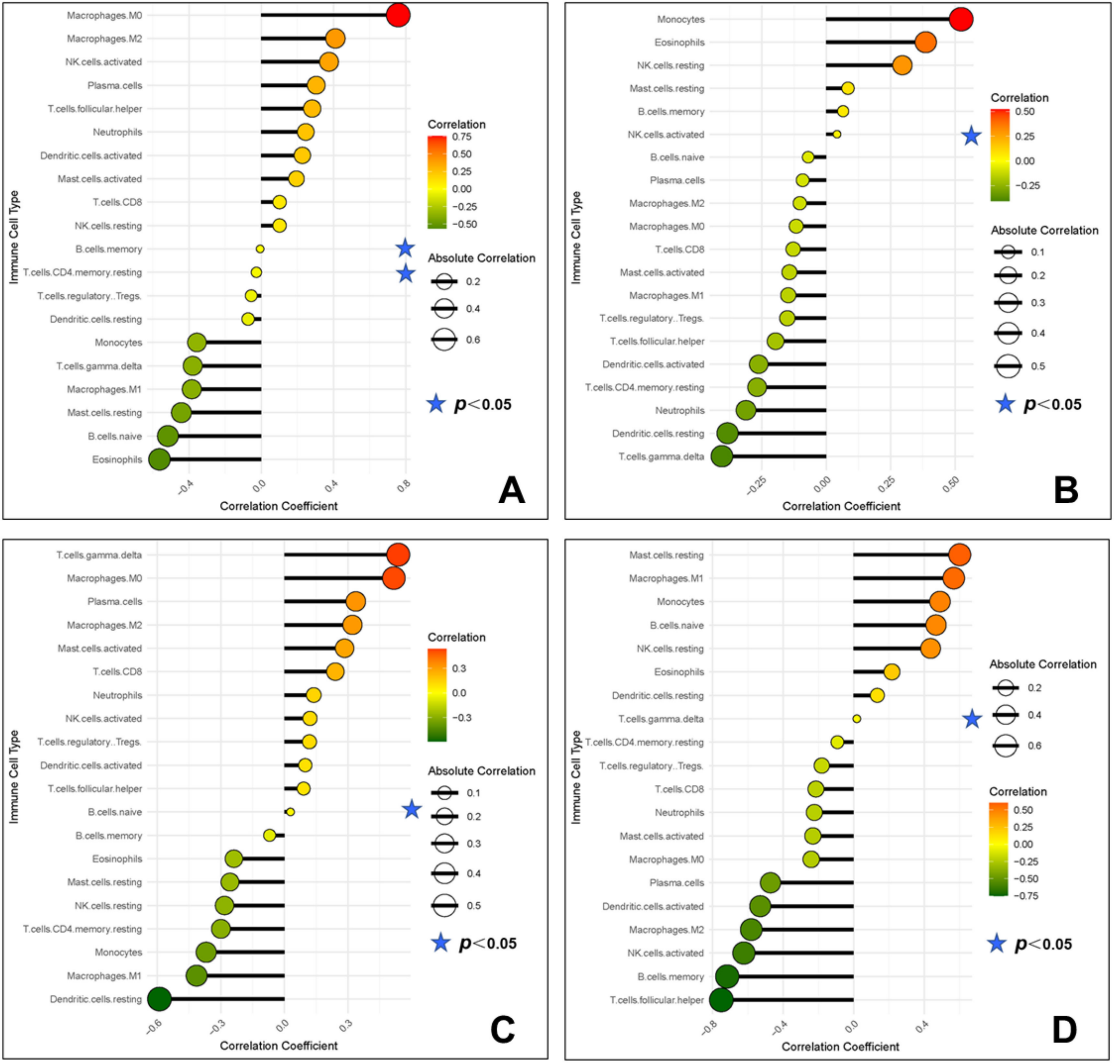
Correlation between gene potential biomarkers and infiltrating immune cells (**A**, WNT5A; **B**, PLCG; **C**, CAMK2A; **D**, ITPR1).

The correlation analysis results showed that WNT5A was negatively correlated with memory B cells and memory CD4+ T cells; PLCG was positively correlated with activated NK cells; CAMK2A was positively correlated with naive B cells; ITPR1 was positively correlated with γδT cells (**Figure 14**).

## 4 Discussion

IRI in the limbs triggers local tissue damage and systemic inflammatory response syndrome (SIRS), significantly impacting patient prognosis (9). Reperfusion following ischemia exacerbates damage beyond ischemia alone, yet the molecular mechanisms remain incompletely elucidated, and effective treatments are limited. Due to the scarcity of limb-specific IRI data, this study utilized model network analysis of cardiac IRI gene expression data to explore shared pathological features and immune infiltration dynamics, offering insights into limb IRI pathogenesis and potential therapeutic targets by leveraging cross-tissue mechanistic similarities.

### 4.1 Enrichment results and intrinsic links to limb IRI

GO functional enrichment analysis of DEGs revealed multiple biological processes and signaling pathways involved in IRI. First, epithelial fluid transport plays a crucial role in maintaining fluid balance and cellular homeostasis; during ischemia, inadequate energy supply disrupts ion pump and channel function, affecting fluid transport, and exacerbating tissue edema and cell damage (10). Second, type II interferons (e.g., IFN-γ) play key roles in inflammation and immune regulation. During IRI, increased IFN-γ expression activates macrophages and T cells, promoting inflammation, but excessive inflammatory responses may exacerbate tissue damage (11). The regulation of fluid levels is crucial for maintaining cellular and tissue functions, as fluid balance systems are activated to restore normal levels when IRI causes increased vascular permeability and fluid leakage leading to edema. Studies (12) have shown that hypoxia response is a core mechanism of IRI, where ischemia leads to insufficient oxygen supply, prompting cells to activate hypoxia-inducible factors (HIF) to adapt to the hypoxic environment. HIF regulates the expression of multiple genes, promoting angiogenesis and metabolic reprogramming, but prolonged hypoxia may result in energy depletion and cell death. Angiogenesis also plays a vital role in restoring blood supply to damaged tissues during IRI. Vascular endothelial growth factor (VEGF) and other pro-angiogenic factors are activated to promote new blood vessel formation. However, dysregulated angiogenesis may lead to abnormal vascular structures and impair tissue repair (13). Platelets are essential for hemostasis and wound healing, but endothelial cell damage induced by IRI can activate platelets, forming thrombi and affecting blood flow (14). Additionally, the extracellular matrix (ECM) supports cell structure and function, but dysregulation of ECM degradation and reconstruction during IRI can impede tissue repair. Secretory granules and vesicles play crucial roles in cell communication and material transport, but IRI can affect their formation and release, disrupting intercellular signaling and material exchange, further impacting tissue function and repair (15). The nuclear membrane protects the nucleus and regulates gene expression, and oxidative stress and inflammation from IRI can compromise its integrity, affecting gene expression and cell function. Growth factors are critical in cell proliferation, differentiation, and repair, and changes in growth factor activity during IRI can influence tissue regeneration and repair; excessive or insufficient growth factor signaling may lead to abnormal tissue responses. Molecular isolation mechanisms regulate the intracellular environment and prevent the spread of damaging factors. IRI leads to cell damage and death, breaking these isolation mechanisms and spreading damaging factors, exacerbating tissue injury. Cytokines are key in regulating immune responses and inflammation. During IRI, cytokine release increases, activating inflammatory responses. While this response helps clear damage and pathogens, excessive cytokine responses may cause inflammatory injury (16).

KEGG analysis results showed that fluid shear stress, relaxin signaling, and calcium signaling pathways have important intrinsic connections in ischemia-reperfusion injury. Fluid shear stress is the tangential force exerted by blood flow on vascular endothelial cells and plays a key role in maintaining endothelial cell function and structural integrity (17). Under normal physiological conditions, stable shear stress promotes anti-inflammatory and antithrombotic properties of endothelial cells. However, during ischemia-reperfusion, sudden restoration of blood flow causes drastic changes in shear stress, leading to endothelial cell activation and damage. Studies have shown that changes in shear stress can activate endothelial inflammation and increase the expression of cell adhesion molecules like VCAM-1 and ICAM-1, promoting atherosclerosis development. Relaxin is a polypeptide hormone that regulates vascular function by activating multiple signaling pathways, including cAMP, NO, and calcium signaling, via its receptor (RXFP1). Relaxin signaling plays an essential role in regulating vascular tension, reducing fibrosis, and mitigating inflammatory responses (18). During IRI, relaxin can reduce ischemic injury by promoting vasodilation and improving microcirculation. It also inhibits the expression of fibrosis-related genes, reducing fibrosis during reperfusion. Calcium ions (Ca^2+^) are critical second messengers within cells, regulating various cellular functions, including contraction, secretion, and metabolism (19). Studies have indicated that dysregulation of calcium signaling is a primary cause of cell damage and death during IRI. Ischemia leads to ATP depletion, impairing calcium pump function and causing intracellular calcium overload. Upon reperfusion, oxidative stress further exacerbates calcium overload, activating calcium-dependent proteases (e.g., calpain) and phospholipases, disrupting cell structure and function. Additionally, calcium overload can induce mitochondrial dysfunction, resulting in apoptosis and necrosis.

### 4.2 Intrinsic links between key genes WNT5A, PLCG, ITPR1, CAMK2A, and limb IRI

This study identified 116 upregulated genes and 53 downregulated genes from microarray analysis. Machine learning analysis further screened out four key genes related to the IRI pathogenesis (WNT5A, PLCG, ITPR1, CAMK2A), which were validated through animal experiments. WNT5A, a critical member of the Wnt signaling pathway, is involved in regulating cell differentiation, proliferation, and migration. In skeletal muscle IRI, the expression of WNT5A is significantly upregulated, indicating its role in inflammation and apoptosis regulation. Research suggests that WNT5A activates calcium signaling through the non-canonical Wnt/Ca^2+^ pathway, causing calcium release from the endoplasmic reticulum into the cytoplasm, further activating calcium-dependent proteases (e.g., calpain) to promote the release of inflammatory mediators and apoptosis. Additionally, WNT5A regulates angiogenesis and fibrosis, influencing tissue repair and regeneration after injury (20). For example, WNT5A promotes endothelial cell migration and proliferation, enhancing new blood vessel formation to improve blood supply to ischemic tissues. PLCG is a crucial signal transduction molecule responsible for transmitting extracellular signals to the intracellular environment, triggering various physiological responses. PLCG hydrolyzes phosphatidylinositol-4,5-bisphosphate (PIP2) to produce diacylglycerol (DAG) and inositol trisphosphate (IP3), where IP3 binds to its receptor on the endoplasmic reticulum membrane, inducing calcium release into the cytoplasm and regulating intracellular calcium signaling. In skeletal muscle IRI, PLCG activation increases intracellular calcium concentration, leading to calcium overload, which induces apoptosis and necrosis (21). Moreover, PLCG activates the PKC pathway, promoting the release of inflammatory factors and exacerbating inflammation. ITPR1 is a calcium release channel on the endoplasmic reticulum membrane that regulates intracellular calcium release. In skeletal muscle IRI, excessive activation of ITPR1 leads to a rapid increase in intracellular calcium, triggering calcium-dependent enzymes such as calpain and phospholipase A2, causing damage to cell membranes and cytoskeleton. Additionally, abnormal ITPR1 activation can lead to mitochondrial calcium overload, inducing oxidative stress and apoptosis. Research (22) suggests that ITPR1 plays a pivotal role in calcium signaling regulation, making it a critical molecule in cell survival regulation. CAMK2A is a key regulatory enzyme in the calcium signaling pathway that regulates various cellular functions. In skeletal muscle IRI, CAMK2A modulates cell growth, metabolism, and apoptosis by phosphorylating downstream effectors. Studies indicate that CAMK2A exerts protective effects by regulating mitochondrial function and reducing oxidative stress (23). Specifically, CAMK2A regulates mitochondrial calcium channels to prevent excessive calcium accumulation, protecting mitochondrial function and reducing cell death. However, overactivation of CAMK2A may also lead to calcium overload and cell death, suggesting its dual role in IRI.

### 4.3 Analysis of immune infiltration results

During limb IRI, changes in the numbers of different types of immune cells reflect complex pathological mechanisms and immune responses. Specifically, the levels of plasma cells, T cells follicular helper, macrophages M0, and macrophages M2 decreased, while macrophages M1, dendritic cells resting, and mast cells activated increased, collectively contributing to the onset and progression of IRI. Plasma cells, which differentiate from B cells, mainly function to secrete large amounts of antibodies (24). During ischemia-reperfusion injury, the deterioration of the local microenvironment and persistent inflammation suppress plasma cell survival and function. The hypoxic conditions induced by ischemia and oxidative stress during reperfusion damage plasma cells, reducing their numbers. Furthermore, the continuous inflammatory response through the secretion of pro-inflammatory cytokines (e.g., TNF-α and IL-6) further inhibits plasma cell survival and function. T follicular helper (Tfh) cells play crucial roles in regulating B cell activation and antibody production. The reduction in Tfh cells during IRI may be due to T cell exhaustion caused by high-intensity inflammation and tissue damage. Strong oxidative stress and inflammation induced by reperfusion consume many Tfh cells, reducing their numbers and functions. Additionally, Tfh cells are sensitive to cytokines and chemokines in the local environment, and changes in these signals may also contribute to the reduction of Tfh cells (25). Macrophages M0 can polarize into pro-inflammatory M1 or anti-inflammatory M2 macrophages in response to local microenvironment signals (26, 27). During IRI, the decrease in M0 macrophages may be associated with their rapid polarization into M1 and M2 macrophages. Tissue damage and inflammatory signals caused by ischemia and reperfusion rapidly activate M0 macrophages, polarizing them into M1 or M2 to respond to injury and initiate repair (28). M2 macrophages have anti-inflammatory and tissue repair functions. However, although M2 macrophages play a crucial role in the later stages of tissue repair during IRI, the early strong inflammatory response and high levels of pro-inflammatory cytokines (e.g., TNF-α and IL-6) may inhibit M2 macrophage production and function. Oxidative stress and tissue damage induced by reperfusion may impair the anti-inflammatory effects of M2 macrophages and increase the proportion of M1 macrophages to balance local immune responses. Unlike the cells mentioned above, M1 macrophages are the primary pro-inflammatory effector cells. During ischemia-reperfusion injury, M1 macrophages participate in the pathological process by secreting high levels of pro-inflammatory cytokines (e.g., TNF-α, IL-1β, and IL-6) and producing reactive oxygen species (ROS). M1 macrophages help clear necrotic tissue and pathogens in the early stages of IRI, but their overactivation may lead to secondary damage and tissue injury. Resting dendritic cells play a key role in immune surveillance and antigen presentation. Dendritic cells can capture and process antigens and present them to T cells to initiate a specific immune response. In skeletal muscle IRI, dendritic cell activation can amplify the inflammatory response by inducing T and B cell responses and regulating other immune cell migration and functions through cytokine and chemokine secretion (29). This process is crucial for the initial immune response and long-term tissue repair. Activated mast cells are key cells involved in acute inflammation and allergic reactions. In skeletal muscle IRI, mast cells rapidly induce vasodilation, increased permeability, and local inflammation by releasing histamine, tryptase, and other mediators. These mediators promote leukocyte exudation and migration, exacerbating local inflammation and tissue damage (30). Although mast cells play a vital role in the initial immune response, their overactivation may lead to excessive inflammation and tissue injury.

### 4.4 Correlation between key genes and immune cell infiltration

During IRI, immune cell infiltration and functional regulation play critical roles in damage progression and repair. Our study found that certain key genes play crucial roles in regulating immune cell infiltration: the key genes WNT5A, PLCG, CAMK2A, and ITPR1 showed positive or negative correlations with specific types of immune cells, reflecting their significant roles in regulating immune cell infiltration and function. The WNT5A gene is negatively correlated with memory B cells and memory CD4+ T cells during IRI. WNT5A is a key member of the Wnt signaling pathway, widely involved in cell differentiation, migration, and tissue repair. Its negative correlation may be due to enhanced local inflammation during ischemia and reperfusion, leading to upregulated WNT5A expression, which suppresses the survival and function of memory B and memory CD4+ T cells. High levels of WNT5A may inhibit these memory cells’ infiltration into damaged tissues by regulating cytoskeletal and migration-related signaling pathways, preventing excessive immune responses that could cause further tissue damage (31). The PLCG gene is positively correlated with NK cell activation. PLCG plays a key role in multiple cell signaling pathways, particularly in regulating immune cell function. PLCG participates in receptor-mediated signal transduction, regulating calcium release and PKC activation, enhancing NK cell activity and cytotoxic function. During IRI, upregulated PLCG expression may promote NK cell activation and enhanced function, allowing more efficient clearance of damaged or degenerated cells, thereby playing a crucial role in the early immune response (32). The CAMK2A gene is positively correlated with naive B cells. CAMK2A is a critical regulator of intracellular calcium signaling, essential for multiple cellular functions. Calcium signaling is vital for B cell proliferation, differentiation, and antibody production. CAMK2A promotes naive B cell activation and enhanced function by regulating intracellular calcium levels. During IRI, upregulated CAMK2A expression may enable naive B cells to play a more significant role in the early immune response, helping the body quickly respond to injury (33). The ITPR1 gene is positively correlated with γδT cells. ITPR1 is a key receptor that regulates intracellular calcium release and participates in various cell functions (22). Calcium signaling is crucial for γδT cell activation and function. γδT cells are a special class of T cells in the immune system with rapid response capabilities to infection and injury. ITPR1 enhances γδT cell activity and function by regulating intracellular calcium release. During IRI, upregulated ITPR1 expression may promote γδT cell activation, enabling a rapid and robust response in the early immune reaction, contributing to early damage control and repair. Studying these genes not only aids in understanding the pathological processes of IRI but also provides a theoretical basis for developing new therapeutic strategies.

### 4.5 Limitations and future work

This study, while offering valuable insights into limb IRI using cardiac IRI gene expression data (GSE36073) and rat models, has several significant limitations that merit detailed discussion. The GSE36073 dataset, derived from CTGF-stimulated adult cardiac myocytes rather than limb IRI samples, was chosen due to the scarcity of high-quality limb-specific datasets in GEO. Our approach is justified by substantial evidence supporting shared inflammatory and immune pathways between cardiac and limb IRI (3, 4), but tissue-specific differences undoubtedly exist and may influence the interpretation of our findings.

A critical methodological limitation of our study is the absence of objective blood flow measurements during the ischemia and reperfusion periods. We acknowledge that Doppler ultrasonography or laser speckle contrast imaging would have provided quantitative verification of blood perfusion cessation during ischemia and restoration during reperfusion. This objective validation would have standardized injury severity across experimental animals and enhanced reproducibility. Our visual assessment of limb pallor and post-ischemic hyperemia, while established in the literature, lacks the precision offered by these advanced imaging techniques. The variability in ischemic severity that may have resulted from this limitation could potentially influence gene expression patterns and immune cell recruitment dynamics.

Another significant limitation is the lack of immunohistochemical validation of the immune cell infiltration patterns predicted by our computational CIBERSORT analysis. While CIBERSORT provides valuable insights into immune cell composition based on gene expression signatures, direct visualization and quantification of specific immune cell populations using immunohistochemical staining would have provided more definitive evidence of immune cell dynamics during IRI. The predicted changes in macrophage polarization (M0, M1, M2), dendritic cells, mast cells, and lymphocyte populations require histological confirmation with specific cellular markers to strengthen our conclusions about immune involvement in limb IRI.

Perhaps most importantly, we recognize that our study lacks functional validation of the identified hub genes (WNT5A, PLCG, ITPR1, CAMK2A). While we have demonstrated their differential expression and correlation with immune cell populations, we have not established causal relationships between these genes and IRI pathophysiology. Gene knockdown or overexpression experiments would have provided direct evidence of their roles in the inflammatory response, cell death, and tissue repair processes during limb IRI. This represents a fundamental limitation of our current work, as functional validation is essential for translating correlative findings into mechanistic insights with therapeutic potential.

To address these constraints, we propose a comprehensive roadmap for future research that would substantively advance this field:

First, we will implement standardized protocols for objective assessment of limb ischemia and reperfusion using Doppler ultrasonography or laser speckle contrast imaging. This will enable precise quantification of blood flow cessation during ischemia and restoration during reperfusion, ensuring consistent injury severity across experimental subjects. These measurements will be correlated with molecular and cellular responses to establish relationships between perfusion dynamics and pathological processes.

Second, to validate and extend our computational predictions of immune cell infiltration, we will conduct extensive immunohistochemical analyses of limb tissues following IRI. This will include staining for markers of macrophages (CD68, CD80 for M1, CD206 for M2), neutrophils (MPO), T-cell subsets (CD3, CD4, CD8), B cells (CD19), dendritic cells (CD11c), and mast cells (tryptase, c-Kit). Multiplex immunofluorescence will allow simultaneous visualization of multiple immune cell populations, providing spatial and temporal information about their interactions during the course of IRI.

Third, to establish the functional roles of our identified hub genes, we plan to conduct *in vitro* and *in vivo* experiments using both loss-of-function and gain-of-function approaches. For *in vitro* studies, we will use RNA interference (siRNA or shRNA) to knockdown each hub gene in skeletal muscle cells subjected to hypoxia-reoxygenation, measuring effects on cell viability, inflammatory cytokine production, and oxidative stress. Complementary overexpression studies using plasmid vectors will determine whether increased expression of these genes is protective or detrimental. For *in vivo* validation, we will utilize adeno-associated virus (AAV) vectors for local delivery of gene-specific shRNA or overexpression constructs to rat limbs prior to IRI induction, assessing effects on tissue damage, inflammatory infiltration, and functional recovery.

Finally, recognizing the limitations of cross-tissue extrapolation, future studies will prioritize the collection and analysis of human limb tissue samples from patients undergoing procedures involving controlled ischemia (e.g., tourniquet application during orthopedic surgery) or from patients with acute limb ischemia. This direct approach will overcome the cross-tissue limitations of the current study and provide clinically relevant insights into human limb IRI pathophysiology.

## 5 Conclusion

This study employed a cross-tissue approach, integrating well-characterized cardiac IRI data (GSE36073) with a rat limb IRI model to systematically identify key genes and mechanisms underlying IRI. By leveraging shared inflammatory pathways, we constructed a biological network model from cardiac data and identified WNT5A, PLCG, ITPR1, and CAMK2A as pivotal regulators of limb IRI pathogenesis. Immune infiltration analysis further revealed a distinct immune landscape, characterized by decreased plasma cells, follicular helper T cells, M0 macrophages, and M2 macrophages, alongside increased M1 macrophages, resting dendritic cells, and activated mast cells. Correlation analyses indicated that WNT5A suppresses memory B and CD4+ T cell survival, PLCG enhances NK cell activation, CAMK2A promotes naïve B cell function, and ITPR1 correlates with γδT cell activity—highlighting immune responses as critical therapeutic targets.

The observed activation of the WNT5A-PLC-CaMKII axis in our model aligns with established inflammatory pathways (**Figures 11**, **12**). While direct measurement of inflammatory cytokines (e.g., IL-6, TNF-α) was not performed, previous studies have demonstrated that this signaling cascade potently induces pro-inflammatory cytokine production via NF-κB activation (8, 34). Histological evidence from the Model Group (**Figure 13**) further supports this mechanism, suggesting that inflammatory cascades are conserved across cardiac and limb IRI models.

HE staining revealed generalized inflammation, including karyorrhexis and granulocyte clusters, but lacked specificity for immune subsets. Future studies should incorporate immunohistochemistry (IHC) with CD68+ (macrophages) and MPO+ (neutrophils) markers to spatiotemporally characterize immune cell dynamics, as outlined in our roadmap (Section 4.6). Despite these limitations, our HE/qPCR data provide mechanistic insights into the “calcium dysregulation → inflammation amplification” axis, with WNT5A emerging as a potential link between calcium signaling and immune activation. Previous work has shown that WNT5A promotes M1 macrophage polarization via NF-κB (34), a process likely exacerbated in ischemic tissues, suggesting a dual role in both calcium-mediated cytotoxicity and immune modulation.

Although pharmacological inhibition (Box5/U-73122) effectively reduced target protein expression (**Figure 11**), potential off-target effects cannot be excluded. To refine our findings, future studies will employ AAV9-shWNT5A delivery for cell-specific gene silencing and single-cell RNA sequencing to map cell type–specific responses.

Through interdisciplinary collaboration and methodological innovation, this study advances the potential of cross-tissue analysis for IRI prevention and treatment, offering new clinical prospects for patients with limb ischemic conditions. Despite its limitations, our findings provide a valuable framework for future investigations into the molecular mechanisms of limb IRI.

## Data Availability

The datasets analyzed for this study can be found in the Gene Expression Omnibus (GEO): GSE36073.
