# Supplementary material for: Identification of key genes and immune infiltration mechanisms in limb ischemia-reperfusion injury: a bioinformatics and experimental study
Source: Front Immunol. 2025 May 9;16:1491928. doi: 10.3389/fimmu.2025.1491928 (PMC12098047; doi:10.3389/fimmu.2025.1491928)
Supplement: Supplementary file 1 [file DataSheet1.docx]

**Supplementary Materials**

**Supplementary Materials: Proposed Follow-Up Study Design for Hub Gene Knockdown/Inhibition**

**Title**: Proposed Experimental Design for Functional Validation of Hub Genes in Limb IRI

**Objective**: To investigate the causal roles of hub genes (*WNT5A*, *PLCG*, *ITPR1*, *CAMK2A*) identified from GSE36073 bioinformatics analysis in limb IRI pathogenesis through siRNA-mediated knockdown, assessing impacts on Wnt/calcium signaling, inflammation, and tissue damage.
**Rationale**: Our study identified *WNT5A*, *PLCG*, *ITPR1*, and *CAMK2A* as hub genes upregulated in GSE36073 and validated in a rat limb IRI model. These genes are implicated in non-canonical Wnt signaling (*WNT5A*), phospholipase C-mediated signal transduction (*PLCG*), intracellular calcium release (*ITPR1*), and calcium-dependent kinase activity (*CAMK2A*), all potentially driving IRI-related inflammation and cell death. Knockdown experiments will elucidate their functional contributions and therapeutic potential.

**Materials and Methods**:

1. **Cell Model**:
   - **Cell Line**: C2C12 mouse myoblasts (skeletal muscle-relevant, responsive to Wnt/calcium signaling).
   - **IRI Simulation**: Hypoxia/reoxygenation (H/R) model—cells cultured in hypoxic conditions (1% O₂, 24h) followed by reoxygenation (21% O₂, 24h).
   - **Control**: Normoxic conditions (21% O₂).
2. **siRNA Knockdown**:
   - **Target Genes**: *WNT5A*, *PLCG1* (or *PLCG2* if intended, clarify), *ITPR1*, *CAMK2A*.
   - **Reagents**: Gene-specific siRNAs (e.g., Silencer Select, Thermo Fisher) and scrambled siRNA (negative control).
   - **Transfection**: Lipofectamine 3000 (Invitrogen) at 50 nM siRNA concentration, optimized for 70–80% knockdown efficiency (verified by qPCR).
   - **Timeline**: Transfect 24h prior to H/R induction.
3. **Experimental Groups**:
   - (1) Normoxia + scrambled siRNA.
   - (2) H/R + scrambled siRNA.
   - (3) H/R + *WNT5A* siRNA.
   - (4) H/R + *PLCG* siRNA.
   - (5) H/R + *ITPR1* siRNA.
   - (6) H/R + *CAMK2A* siRNA.
   - (7)H/R + combined siRNA (e.g., *WNT5A* + *ITPR1*) to explore pathway interactions.
4. **Outcome Measures**:
   - **Gene Expression**: RT-qPCR to confirm knockdown (*WNT5A*, *PLCG*, *ITPR1*, *CAMK2A*) and downstream targets (e.g., *FZD2* for Wnt, *IP3* signaling genes, *CREB* for CAMK2A).
   - **Protein Levels**: Western blot for WNT5A, PLCG1 (phosphorylated), ITPR1, and CAMK2A (phospho-Thr286), normalized to β-actin.
   - **Calcium Signaling**: Fluo-4 AM staining and fluorescence microscopy to measure intracellular Ca²⁺ levels post-H/R.
   - **Inflammation**: ELISA for cytokines (e.g., IL-6, TNF-α) linked to Wnt/calcium pathways.
   - **Cell Viability**: MTT assay to assess protection against H/R-induced damage.
   - **Immune Response**: Flow cytometry for macrophage co-culture (e.g., RAW 264.7 cells) to evaluate infiltration markers (CD11b, F4/80).
5. **Animal Model Validation**:
   - **Model**: Rat limb IRI (60 min ischemia, 24h reperfusion, as per main study).
   - **Intervention**: Intra-muscular injection of siRNA-loaded nanoparticles (e.g., chitosan-based) targeting *WNT5A*, *PLCG1*, *ITPR1*, and *CAMK2A*, 48h pre-ischemia.
   - **Controls**: Scrambled siRNA and saline.
   - **Outcomes**:
     - Histology (H&E staining) for tissue damage.
     - Immunohistochemistry for macrophage infiltration (F4/80) and Wnt/calcium markers (e.g., phospho-CAMK2A).
     - qPCR/Western blot for gene/protein expression and pathway activity.
6. **Statistical Analysis**:
   - One-way ANOVA with post-hoc Tukey’s test (n = 3–6 per group).
   - Significance threshold: p < 0.05.

**Expected Outcomes**:

- **WNT5A Knockdown**: Reduced non-canonical Wnt signaling (e.g., decreased *FZD2* expression), lowering inflammation (e.g., 20–30% reduced IL-6) and improving cell viability.
- **PLCG Knockdown**: Decreased PLC activity, reducing downstream IP3/Ca²⁺ release, attenuating oxidative stress and cytokine production.
- **ITPR1 Knockdown**: Lowered Ca²⁺ release from ER, reducing CAMK2A activation and apoptosis (e.g., 25% increased viability).
- **CAMK2A Knockdown**: Diminished Ca²⁺-dependent kinase activity (e.g., reduced phospho-CREB), decreasing inflammation and necrosis.
- **In Vivo**: Reduced tissue damage and immune infiltration in siRNA-treated rats, with synergistic effects from combined knockdown.

**Potential Therapeutic Implications**:

- Validating *WNT5A*, *PLCG1*, *ITPR1*, and *CAMK2A* as IRI drivers could guide targeted therapies:
  - *WNT5A*: Wnt pathway inhibitors (e.g., Box5).
  - *PLCG*: PLC inhibitors (e.g., U73122).
  - *ITPR1*: IP3 receptor blockers (e.g., 2-APB).
  - *CAMK2A*: CAMK inhibitors (e.g., KN-93).
- These could mitigate IRI in clinical contexts like limb reperfusion or transplantation by reducing calcium-mediated damage and inflammation.

**Limitations and Considerations**:

- Off-target siRNA effects will be assessed via RNA-seq.
- Cell line responses may differ from in vivo skeletal muscle; animal validation will address this.
- Pathway crosstalk (e.g., Wnt/Ca²⁺ overlap) may complicate single-gene interpretations, necessitating combined knockdown analysis.
